# Post-traumatic stress disorder and restraint in patients followed at Arrazi Hospital

**DOI:** 10.1192/j.eurpsy.2023.2082

**Published:** 2023-07-19

**Authors:** L. Azizi, O. Seyar, N. Baabouchi, F. Laboudi, A. Ouanass

**Affiliations:** Hôpital Arrazi Salé, Salé, Morocco

## Abstract

**Introduction:**

Post-traumatic stress disorder (PTSD) is characterized by intense, unpleasant, and dysfunctional reactions that appear after an overwhelming traumatic event. A life-threatening or serious injury event can cause lasting and intense suffering.

Hospitalization without consent, the use of isolation techniques, restraint or the obligation to take treatment are situations that can be perceived as traumatic.

**Objectives:**

Our goal is: The search for the existence of psycho-pathological links and vulnerabilities between the state of post-traumatic stress and physical restraint. Consideration of the traumatic event in the development of appropriate care strategies.

Our goal is:The search for the existence of psycho-pathological links and vulnerabilities between the state of post-traumatic stress and physical restraint.Consideration of the traumatic event in the development of appropriate care strategies.

**Methods:**

To meet this objective, we carried out a descriptive study which identified 200 cases collected in the psychiatric emergency department of the Arrazi Hospital in Salé from 2017 to 2021. An exploitation sheet was drawn up and the study was carried out using Meta-chart and Visuel-chart software.

**Results:**

At the end of this work we found: The average age is 35 years +/- the sex ratio is equal to 1.42. 80% are single while 10% are divorced. Regarding professional activity, 70% are unemployed. 10% of patients have a level of education above the baccalaureate.

As for the history, 60% of the cases studied have a personal psycho-addictive history. 34.6% of the cases studied have a medical-surgical history.

The psychiatric pathologies that were found are: 10% suffer from depressive disorder, 80% have psychosis, 10% have attempted suicide.

**Conclusions:**

During an acute episode, patients may be exposed to coercive treatments. Hospitalization without consent, the use of isolation techniques, restraint or the obligation to take treatment are all factors that can be perceived as traumatic. For example, patients recently exposed to a first psychotic episode confirmed the traumatic character of the psychotic episode itself, of the treatment or of both.

**Disclosure of Interest:**

None Declared

